# Genetic Determinants of Parkinson's Disease: Can They Help to Stratify the Patients Based on the Underlying Molecular Defect?

**DOI:** 10.3389/fnagi.2017.00020

**Published:** 2017-02-10

**Authors:** Sara Redenšek, Maja Trošt, Vita Dolžan

**Affiliations:** ^1^Pharmacogenetics Laboratory, Institute of Biochemistry, Faculty of Medicine, University of LjubljanaLjubljana, Slovenia; ^2^Department of Neurology, University Medical Centre LjubljanaLjubljana, Slovenia

**Keywords:** Parkinson's disease, genetic risk factors, genome-wide association studies, single nucleotide polymorphisms, personalized medicine

## Abstract

Parkinson's disease (PD) is a sporadic progressive neurodegenerative brain disorder with a relatively strong genetic background. We have reviewed the current literature about the genetic factors that could be indicative of pathophysiological pathways of PD and their applications in everyday clinical practice. Information on novel risk genes is coming from several genome-wide association studies (GWASs) and their meta-analyses. GWASs that have been performed so far enabled the identification of 24 loci as PD risk factors. These loci take part in numerous cellular processes that may contribute to PD pathology: protein aggregation, protein, and membrane trafficking, lysosomal autophagy, immune response, synaptic function, endocytosis, inflammation, and metabolic pathways are among the most important ones. The identified single nucleotide polymorphisms are usually located in the non-coding regions and their functionality remains to be determined, although they presumably influence gene expression. It is important to be aware of a very low contribution of a single genetic risk factor to PD development; therefore, novel prognostic indices need to account for the cumulative nature of genetic risk factors. A better understanding of PD pathophysiology and its genetic background will help to elucidate the underlying pathological processes. Such knowledge may help physicians to recognize subjects with the highest risk for the development of PD, and provide an opportunity for the identification of novel potential targets for neuroprotective treatment. Moreover, it may enable stratification of the PD patients according to their genetic fingerprint to properly personalize their treatment as well as supportive measures.

## Introduction

Parkinson's disease (PD) is the second most common neurodegenerative brain disease after Alzheimer's disease. It is believed that both genetic and environmental factors contribute to the development of PD. It is a chronic, progressive and incurable disease (Pringsheim et al., 2014). Two pathological hallmarks characterize PD: the formation of cytoplasmic inclusions termed Lewy bodies and Lewy neurites and the loss of dopaminergic neurons in the substantia nigra pars compacta (Gallegos et al., 2015). The disease manifests as bradykinesia, muscular rigidity, rest tremor and postural and gait impairment (Postuma et al., 2015), which can be accompanied by several other nonmotor symptoms (Gallegos et al., 2015). At the present time, there is unfortunately no cure for PD except for the symptomatic treatment and supportive measures (Connolly and Lang, 2014).

Two different forms of PD have been identified, i.e., sporadic and familial. The familial form starts at an earlier stage of life (<50 years), is usually more severe and progresses more quickly (Klein and Westenberger, 2012). At early stages of research on genetics of PD, only linkage studies were performed, which means that families with PD history were included in the study. These studies identified mutations in six genes that conclusively cause monogenic PD—*SNCA, LRRK2, Parkin, PINK1, DJ-1, ATP13A2* (Klein and Westenberger, 2012).

In this review, we focus mainly on the sporadic form of the disease. However, there is some overlap between genes associated with familial and sporadic disease; in particular, *SNCA* and *LRRK2* are involved in both forms (Verstraeten et al., 2015; van der Brug et al., 2015). Recently, genome-wide association studies (GWASs) that compared single nucleotide polymorphisms' (SNPs) frequencies between sporadic PD patients and healthy individuals have identified several loci as Parkinson's disease susceptibility genes (Klein and Westenberger, 2012; Nalls et al., 2014). A few of these loci, 24 in particular, harboring SNPs that were found to be associated with PD were validated in the replication phase of the latest and largest meta-analysis of GWASs performed by Nalls et al. in 2014. In the discovery phase, they performed a meta-analysis of GWASs including 13,708 cases and 95,282 controls chosen from the populations of European descent (the USA, France, Germany, Iceland, the Netherlands, the UK; 23 and Me), while the replication phase of the study included 5,353 cases and 5,551 controls (Nalls et al., 2014). The identified loci segregate with numerous cellular pathways that may contribute to PD pathology: protein aggregation, protein, and membrane trafficking, lysosomal autophagy, immune response, synaptic function, endocytosis, inflammation, and metabolic pathways being among the most important ones (Kumaran and Cookson, 2015). Each locus identified as a risk factor has a rather low contribution to PD development; therefore, a combination of molecular defects rather than a single event probably plays a role in PD risk, hence the idea of a cumulative nature of genetic risk factors (Pihlstrom et al., 2016).

This review summarizes the latest knowledge on genetics and genomics of PD susceptibility, obtained by GWASs and their meta-analyses. We focus on the largest meta-analysis of GWASs on PD risk so far (Nalls et al., 2014), but we also searched the PubMed database and GWAS catalog (Welter et al., 2014) for studies that pointed out the same loci as the above-mentioned meta-analysis. We identified 13 GWASs and their meta-analyses (summarized in Table 1), which we included in this review. We compiled the available data on the 24 loci that were found to be associated with the risk of sporadic form of PD. To obtain the information about gene functions, we searched the PubMed database and collected the available data on genes' functions with the help of the following words: “Parkinson's disease and *gene name*” or “Parkinson's disease and polymorphisms and *gene name*.” We divided the susceptibility genes into seven groups according to their physiological functions: protein aggregation; protein, and membrane trafficking; lysosomal autophagy; immune system; neurodevelopment, neuron cell differentiation, and survival; mitochondrial homeostasis; and genes involved in other processes. This review highlights the main functions of these genes' products and their role in the PD pathogenesis.

**Table 1:** **GWAS studies and their meta-analyses that identified 24 risk loci included in this review**.

**Study**	**Ancestry**	**Number of cases**	
		**Initial phase (cases vs. controls)**	**Replication phase (cases vs. controls)**
Nalls et al., 2014^#^	European Initial phase—^*^USA, ^*^France, ^*^Germany, ^*^Iceland, ^*^Netherlands, ^*^UK, ^*^23andMe Replication phase—^*^US, ^*^UK, ^*^France, ^*^Germany, ^*^Greece, ^*^Netherlands, ^*^NeuroX	13,708 vs. 95,282	5,353 vs. 5,551
Vacic et al., 2014	Ashkenazi Jewish Initial phase—^*^Israel, ^*^US Replication phase—^*^Israel, ^*^US	1,130 vs. 2,611	306 vs. 2,583
Hill-Burns et al., 2014	European Initial phase—^*^US Replication phase—not reported	1,565 sporadic cases + 435 familial cases vs. 1,986 controls	1,528 sporadic cases + 707 familial cases vs. 796 controls
Lill et al., 2012^#^	Initial phase—^*^US Replication phase—European and Asian	2,197 vs. 2,061	Up to 98,080
Pankratz et al., 2012^#^	European	4,238 vs. 4,239	3,738 vs. 2,111
Do et al., 2011	European	3,426 vs. 29,624	Not applicable
Nalls et al., 2011^#^	Initial phase—^*^France, ^*^Germany, ^*^UK, ^*^US Replication phase—^*^US, ^*^France, ^*^Germany, ^*^Netherlands, ^*^Iceland, ^*^UK	5,333 vs. 12,019	7,053 vs. 9,007
Hamza et al., 2010	European	2,000 vs. 1,986	Up to 1,447 vs. 1,468
Saad et al., 2011	European	1,039 vs. 1,984	3,232 vs. 7,064
Spencer et al., 2011	Initial phase—^*^UK Replication phase—^*^France	1,705 vs. 5,175	1,039 vs. 1,984
Edwards et al., 2010	European	1,752 vs. 1,745	Not applicable
Satake et al., 2009	Japanese	988 vs. 2,521	933 vs. 15,753
Simon-Sanchez et al., 2009	European	1,713 vs. 3,978	3,361 vs. 4,573

* *country of recruitment, if specified*.

# *meta-analysis*.

*Data compiled from the GWAS catalog database and PDgene (Lill et al., 2012; Nalls et al., 2014; Welter et al., 2014)*.

## Genetic determinants of risk for sporadic PD and their implicated pathways

### Protein aggregation

The products of the genes listed below, i.e., SNCA and tau, are both constituents of the protein aggregates typical of PD called Lewy bodies. PD is often thought to be a prion-like disease because of the presence of these bodies (Hasegawa et al., 2016).

#### SNCA

*SNCA* codes for α-synuclein (SNCA), which is a small, acidic protein of 14.5 kDA and 140 amino acids (Gallegos et al., 2015). It is the main component of aggregates called Lewy bodies, a hallmark of PD pathology. Lewy bodies are formed because a mutated protein usually adopts the β-sheet structure, which is harder to degrade than α-helixes, the main conformation of native proteins (Gallegos et al., 2015; Inoshita and Imai, 2015). Different variants of the gene have been connected to both familial and sporadic forms of PD. Several studies on families with positive PD history have reported the association of *SNCA* with PD, the first one being published in 1997 by Polymeropoulos et al. (1997). The association with sporadic PD was first described by Kruger et al. who compared the allelic frequences of *REP1* polymorphism in the promoter region of *SNCA* between cases and controls (Kruger et al., 1999). This association was later confirmed on a larger set of data (Maraganore et al., 2006). The majority of GWASs performed also confirmed *SNCA* as a susceptibility gene (Satake et al., 2009; Simon-Sanchez et al., 2009; Edwards et al., 2010; Hamza et al., 2010; Do et al., 2011; Nalls et al., 2011, 2014; Saad et al., 2011; Spencer et al., 2011; Lill et al., 2012; Pankratz et al., 2012; Hill-Burns et al., 2014). To date, over 800 SNPs within *SNCA* have been reported, with nearly half of them showing a positive association with PD (Kumaran and Cookson, 2015). SNPs within *SNCA* recognized as PD susceptibility factors by GWASs are listed in Table 2. The most replicated SNP rs256220 was confirmed as a risk factor in six studies, whereas several others were found in only one or two studies. Some *SNCA* mutations can cause both sporadic and familial forms of PD. These are usually more penetrant (Singleton et al., 2013). Besides point mutations—Ala53Thr, Ala30Pro, and Glu46Lys in the amino-terminal sequence, whole locus multiplications (duplications and triplications) were also found in both forms (Bisaglia et al., 2009).

**Table 2:** **SNPs within loci associated with protein aggregation recognized as PD susceptibility factors by GWASs**.

**Gene/locus Chromosome**	**SNP**	**MAF (1000 genomes project)**	**Risk allele: OR**	**Study**
*SNCA* Ch 4	rs356182 g.90626111G>A (intron variant)	G = 0.4044	G: OR = 1.32	Nalls et al., 2014
	rs356219 g.90637601G>A (intron variant)	A = 0.4892	OR = 1.29	Lill et al., 2012
			G: OR = 1.29	Nalls et al., 2011
	rs356220 g.90641340T>C (intron variant)	C = 0.4834	OR = 1.38	Pankratz et al., 2012
			T: OR = 1.29	Do et al., 2011
			T: OR = 1.27	Spencer et al., 2011
			T: OR = 1.38	Hamza et al., 2010
			T: OR = 1.38	Hill-Burns et al., 2014
			T: OR = 1.37	Saad et al., 2011
	rs11931074 g.90639515G>T (intron variant)	T = 0.3806	OR = 1.37	Satake et al., 2009
	rs2736990 g.90678541G>A c.307-28113C>T (intron variant)	A = 0.3934	G: OR = 1.23	Simon-Sanchez et al., 2009
			OR = 1.29	Edwards et al., 2010
	rs6532194 g.90780902C>T (intergenic variant)	T = 0.3694	OR = 1.29	Lill et al., 2012
*MAPT* Ch 8	rs17649553 g.43994648C>T (intron variant)	T = 0.0865	C: OR = 1.3	Nalls et al., 2014
	900 kb inversion	H1/H2		Lill et al., 2012
	rs2942168 g.43714850C>T (intron variant)	A = 0.0863	G: OR = 1.27	Nalls et al., 2011
	rs393152 g.43719143A>G (intron variant)	G = 0.2476	A: OR = 1.3	Simon-Sanchez et al., 2009
	rs12185268 g.43923683A>G p.Ile471Val (missense variant)	G = 0.0863	A: OR = 1.3	Do et al., 2011
	rs8070723 g.44081064A>G c.1828-6612A>G (intron variant)	G = 0.1190	OR = 1.3	Spencer et al., 2011
	rs17577094 g.44187492A>G c.1290-15425T>C (intron variant)	G = 0.0865	OR = 1.56	Vacic et al., 2014
	rs11012 g.43513441C>T c.^*^1783G>A	T = 0.0807	T: OR = 1.43	Edwards et al., 2010
	rs199498 g.46788237T>C c.81-14328A>G (intron variant)	C = 0.2578	T: OR = 1.35	Hill-Burns et al., 2014
	rs199533 g.44828931G>A p.Lys702 = (synonimous variant)	A = 0.0791	C: OR = 1.35	Hamza et al., 2010

*Ch, Chromosome. Data compiled from the GWAS catalog database, the dbSNP and the e!Ensembl (Sherry et al., 2001; Welter et al., 2014; Yates et al., 2016)*.

* *Denotes SNPs in 3'-UTR*.

SNCA is widely expressed in the central nervous system, especially in the presynaptic terminals of neurons (Inoshita and Imai, 2015). It has two main physiological roles, the first being the control of synaptic membrane processes and the second being the control of neurotransmitter release via interactions with members of the SNARE family (Bellucci et al., 2012; Tsigelny et al., 2012). It promotes SNARE-complex assembly through a non-enzymatic mechanism, binding to phospholipids via its N-terminal and to synaptobrevin-2 via its C-terminal (Burre et al., 2010). SNCA is deeply involved in the synaptic vesicle cycle, including trafficking, docking, fusion, and recycling after exocytosis. It was also suggested that SNCA is a negative regulator of neurotransmitter release, including dopamine, with traffic restriction of synaptic vesicles from the resting pool to the sites of release (Emanuele and Chieregatti, 2015).

Wild-type SNCA is translocated to the lysosomes for degradation via chaperone-mediated autophagy via the lysosomal membrane receptor LAMP2A (Gan-Or et al., 2015). Lysosomal enzyme β-glucocerebrosidase (GBA) then modulates SNCA levels (Klein and Westenberger, 2012). Mutant SNCA somehow inhibits chaperone-mediated autophagy, presumably by attaching to the LAMP2A and preventing its internalization of wild-type and mutant SNCA. Recently, it has been shown that GBA and SNCA form a positive feedback loop that leads to the accumulation of SNCA. The loss of GBA function results in the accumulation of SNCA, whereas SNCA inhibits the lysosomal activity of GBA (Mazzulli et al., 2011).

SNCA is presumably also involved in dopamine release and in the synaptic vesicle dynamics, especially in the recycling pathway. The excess of SNCA reduces the recycling of synaptic vesicles and their motility (Inoshita and Imai, 2015). Furthermore, it has been confirmed that dopamine can interact with the SNCA molecule via the C-terminal residues which can induce and/or modulate its structure and oligomerization. As a result dopamine blocks chaperone-mediated autophagy of SNCA. This may explain why dopaminergic neurons are more prone to SNCA accumulation (Bellucci et al., 2012; Lashuel et al., 2013; Gan-Or et al., 2015).

SNCA also modulates dopamine vesicle trafficking by other mechanisms. According to Ahn et al. SNCA binds and consequently inhibits phospholipase D2 (PLD2), which is involved in vesicle trafficking in terms of endo- and exocytosis (Ahn et al., 2002). SNCA's interacting partner is also actin which is essential for synaptic vesicle mobilization between different functional pools. SNCA binding modulates actin's polymerization in a manner dependent on Ca^2+^ concentration (Bellani et al., 2010). Intracellular Ca^2+^ concentration may also change due to the formation of pore-like structures in the cell membranes by mutant SNCA (Lashuel et al., 2002). Furthermore, SNCA's interacting partner is also a rate-limiting enzyme in the dopamine synthesis, tyrosine hydroxylase (TH), responsible for the conversion of tyrosine to L-3,4-dihydroxyphenylalanine (L-DOPA). This interaction inhibits the phosphorylation of TH and thus also its action (Venda et al., 2010).

SNCA is also linked to neuroinflammation. In mice with aggregated SNCA, MHCII expression is induced in microglia. Moreover, SNCA as a danger-associated molecular pattern (DAMP) stimulates toll-like receptors (TLR). Misfolded and fibrillar SNCA activates microglia via TLR2 and TLR4 and activation of NLRP3 inflammasome (Gustot et al., 2015). These processes contribute to dopaminergic neuronal death (Dzamko et al., 2015; Kumaran and Cookson, 2015).

In addition, *SNCA* mutations can lead to dysfunctions in several other cellular pathways, such as mitochondrial function, oxidative stress, cytochrome c release, ER stress, apoptosis (Gallegos et al., 2015).

#### MAPT

*MAPT* encodes a microtubule-associated protein tau, which has a role in stabilizing microtubules in the neurons. The gene was first recognized as a PD risk factor by Pastor et al. (2000). It has been repeatedly confirmed as a risk factor for sporadic PD by GWASs and their meta-analyses (Simon-Sanchez et al., 2009; Edwards et al., 2010; Hamza et al., 2010; Do et al., 2011; Nalls et al., 2011, 2014; Spencer et al., 2011; Lill et al., 2012; Hill-Burns et al., 2014; Vacic et al., 2014), but each variant has only been pointed out once and has not been replicated in other studies. More than 40 mutations have been discovered to date. They are either coding mutations, that affect the binding activity of the tau or its susceptibility to aggregation, or mutations that alter the splicing of exon 10 which encodes the fourth microtubule binding repeat (4R tau isoforms) (Wray and Lewis, 2010). Mutations within *MAPT* locus recognized as PD susceptibility factors by GWASs are listed in Table 2. The *MAPT* locus is the longest region of linkage disequilibrium in Caucasians. There are two variants of the locus resulting from an inversion of a 900 kb long portion of the sequence, which leads to two haplotypes—H1 and H2. *MAPT* H1 haplotype increases the transcription of the gene, whereas *MAPT* H2 haplotype decreases it (Golpich et al., 2015). The inversion is present in the H2 haplotype, which has a 20% frequency in the Caucasians, but is almost not present in the Asian population (Wider et al., 2010; Schulte and Gasser, 2011; Soto-Ortolaza and Ross, 2016). *MAPT* association was not replicated in the Japanese population probably due to inter-population heterogeneity at this locus (Satake et al., 2009; Labbé and Ross, 2014). H1 haplotype is more dynamic and can be subdivided into subhaplotypes. Homozygous *MAPT* H1/H1 genotype is a biomarker of dementia in PD (Lin and Wu, 2015). As H1 haplotype is associated with a greater risk of PD and other neurodegenerative diseases, it may be assumed that H2 haplotype is under positive selection (Schulte and Gasser, 2011). Polymorphisms in the *MAPT* gene are also involved in the pathologies of Alzheimer's disease (AD), progressive supranuclear palsy (PSP), and corticobasal degeneration (CBD) (Vandrovcova et al., 2009).

*MAPT* encoded protein tau is a potential component of Lewy bodies. In PD patients with a more pronounced cognitive decline, neurofibrillary tangles, more typical of AD, can also be found besides Lewy bodies. These tangles consist of hyperphosphorylated tau (Horvath et al., 2013; Lin and Wu, 2015). Physiologically, phosphorylation of the tau protein regulates its propensity to interact with microtubules (Wray and Lewis, 2010). According to some studies, SNCA also promotes tau fibrillization and influences tau phosphorylation (Giasson et al., 2003). The interplay between the two genes was also confirmed by the study of Goris et al., reporting nearly a double risk of PD in carriers of the combined *MAPT* H1/H1 and *SNCA* rs356219 G/G genotype (Goris et al., 2007). Most of the pathological changes in the *MAPT* sequence disrupt the ability of tau to interact with microtubules (Wray and Lewis, 2010). Tau is also fundamental for the maintenance of cytoskeletal network via the regulation of axonal transport by interactions with kinesin and dynein (Kumaran and Cookson, 2015).

Mutated MAPT may also affect the function of lysosomes and its role in autophagy, since it is degraded with this process (Gan-Or et al., 2015).

*MAPT* gene lies in a block of nearly complete linkage disequilibrium that extends over nearly 2 Mb, therefore it is possible that other genes from this region are also associated with PD risk (Charlesworth et al., 2012). For example, a variant in the *CRHR1* gene coding for the corticotropin-releasing hormone receptor has recently been recognized as a factor that decreases the PD susceptibility (Davis et al., 2016).

### Protein and membrane trafficking

SNPs within loci associated with protein and membrane trafficking recognized as PD susceptibility factors by GWASs are listed in Table 3. The genes within this group are involved in processes, such as clathrin-mediated vesicular transport, trafficking, and fusing of synaptic vesicles, clearance of Golgi-derived vesicles, endo- and exocytosis and protein sorting.

**Table 3:** **SNPs within loci associated with protein and membrane trafficking recognized as PD susceptibility factors by GWASs**.

**Gene/locus**	**SNP**	**MAF (1000 genomes project)**	**Risk allele: OR**	**Study**
*TMEM175/GAK/DGKQ* Ch 4	rs34311866 g.951947T>C p.Met311Thr (missense variant)	C = 0.1400	C: OR = 1.27	Nalls et al., 2014
	rs11248060 g.964359C>T (intron variant)	T = 0.1066	OR = 1.21	Lill et al., 2012
			OR = 1.26	Pankratz et al., 2012
			NA	Edwards et al., 2010
	rs6599388 g.939087C>T (intron variant)	T = 0.3243	T: OR = 1.16	Nalls et al., 2011
	rs11248051 g.858332C>T (intron variant)	T = 0.0966	T: OR = 1.46	Hamza et al., 2010
	rs6599389 g.939113G>A (intron variant)	A = 0.1092	A: OR = 1.31	Do et al., 2011
*NUCKS1/RAB7L1* Ch 1	rs823118 g.205723572C>T (upstream gene variant)	T = 0.4111	T: OR = 1.122	Nalls et al., 2014
	rs947211 g.205752665A>G (intergenic variant)	A = 0.4613	OR = 1.3	Satake et al., 2009
	rs823128 g.205713378G>A (intron variant)	G = 0.1765	A: OR = 1.52	Simon-Sanchez et al., 2009
	rs823114 g.205719532G>A c.-431C>T (upstream gene variant)	A = 0.4073	OR = 1.33	Vacic et al., 2014
*LRRK2* Ch 12	rs76904798 g.40614434C>T (intron variant)	T = 0.1318	T: OR = 1.155	Nalls et al., 2014
	rs34637584 g.40734202G>A p.Gly2019Ser (missense variant)	A = 0.0002	A: OR = 9.62	Do et al., 2011
	rs1442190 g.41365640G>A c.^*^325G>A	A = 0.0747	OR = 3.72	Vacic et al., 2014
	rs34778348 g.40757328G>A p.Gly2385Arg (missense variant)	A = 0.0048	OR = 2.23	Lill et al., 2012
	rs1491942 g.40620808C>G c.237+1366C>G (intron variant)	G = 0.2979	OR = 1.17	Lill et al., 2012
			G: OR = 1.27	Nalls et al., 2011
	rs1994090 g.40428561G>T c.717-6250C>A (intron variant)	G = 0.1472	OR = 1.39	Satake et al., 2009
*INPP5F* Ch 10	rs117896735 g.121536327G>A c.179-4820G>A (intron variant)	A = 0.0040	A: OR = 1.624	Nalls et al., 2014
*BCKDK/STX1B* Ch 16	rs14235 g.31110472G>A c.615G>A p.Thr205 = (synonymous variant)	A = 0.3592	A: OR = 1.103	Nalls et al., 2014
*VPS13C* Ch 15	rs2414739 g.61701935G>A (intron variant)	G = 0.3209	A: OR = 1.113	Nalls et al., 2014

*NA, data not available; Ch, Chromosome. Data compiled from the GWAS catalog database, the dbSNP and the e!Ensembl (Sherry et al., 2001; Welter et al., 2014; Yates et al., 2016)*.

* *Denotes SNPs in 3'-UTR*.

#### TMEM175/GAK/DGKQ

A haplotype block *TMEM175/GAK/DGKQ* (transmembrane protein 175/cyclin G associated kinase/theta diacylglicerol kinase) was first associated with sporadic PD in the GWAS performed by Hamza et al. after it was already confirmed to be associated with the familial form of the disease (Pankratz et al., 2009; Hamza et al., 2010). This locus was reevaluated several times by different research groups and also showed positive results in other GWASs and meta-analyses of GWASs. The most replicated SNP is an intron variant rs11248060, others were found in individual studies only (Edwards et al., 2010; Do et al., 2011; Nalls et al., 2011, 2014; Lill et al., 2012; Pankratz et al., 2012).

*GAK* is a particularly promising candidate for a risk gene, because it is differentially expressed in the substantia nigra pars compacta of PD patients as compared to controls (Pankratz et al., 2009). *GAK* gene product participates in multiple steps of clathrin-mediated vesicular transport. For example, together with the heat-shock cognate protein 70 (Hsc70), it promotes the uncoating of endocytosed clathrin-coated vesicles. Probably it has also some other functions related to its serine/threonine kinase domain. It also directly interacts with LRRK2 and participates in the clearance of Golgi-derived vesicles via the lysosomal autophagy pathway as a part of LRRK2/RAB7L1/GAK trans-Golgi complex (Kumaran and Cookson, 2015; Perrett et al., 2015). Recently, depletion of GAK has been shown to influence lysosomal sorting of cathepsin D, the main lysosomal enzyme involved in SNCA degradation (Latourelle et al., 2009). Knockdown of GAK in primary rat neurons increased SNCA toxicity (Perrett et al., 2015). GAK also takes part in cell cycle regulation (Labbé and Ross, 2014).

Less is known about *DGKQ*; however, it is expressed in the brain where it may be involved in the phosphatidylinositol and lipid signaling (Pankratz et al., 2009). DGK proteins are also known to affect Ca^2+^ signaling as well as the trafficking and fusing of synaptic vesicles at nerve terminals in the central nervous system (Ran and Belin, 2014).

#### NUCKS1/RAB7L1

A haplotype block NUCKS1/RAB7L1 (nuclear casein kinase and cyclin-dependent kinase substrate 1/RAB7, member RAS oncogene family-like 1) is in linkage disequilibrium and is also known by the name PARK16 locus. This locus contains five genes—*SLC45A3, NUCKS1, RAB7L1, SLC41A1*, and *PM20D1*. The association between PD and *NUCKS1* was first described in the GWAS conducted by Simon-Sanchez et al. (2009), whereas the association with *RAB7L1* was first shown in Satake et al. GWAS (Satake et al., 2009). The implication of this locus in PD was then again confirmed in meta-analysis of GWASs by Nalls et al. and in the GWAS by Vacic et al. (Nalls et al., 2014; Vacic et al., 2014). Four SNPs were pointed out by genome-wide studies in this locus but none of them was replicated.

RAB7L1, also named RAB29, is a cytoplasmic GTP-binding protein, which plays an important role in endo- and exocytosis. Exocytosis is relevant in PD because dopamine is released via exocytotic vesicles and because it has been shown that vesicle abnormalities occur in SNCA knock-out mice (Plagnol et al., 2011). RAB7L1 is one of the LRRK2 interacting partners in the process of removal of Golgi derived vesicles by autophagy-dependent mechanisms. RAB7L1 deficiency caused neurodegeneration in mammalian or *Drosophila* dopaminergic neurons having a human *LRRK2* mutation, while *RAB7L1* overexpression restored the function of *LRRK2* mutant neurons. MacLeod et al. showed that expressing mutant LRRK2 or reducing the expression of RAB7L1 in a cell line led to the loss of components of the retromer complex, which regulates protein sorting from the lysosome to the Golgi apparatus. Secondary to these changes, the swelling of lysosomes and the reduction of lysosomal mannose 6-phosphate receptor (M6PR) accumulation occur (MacLeod et al., 2013; Perrett et al., 2015). M6PRs are needed for the recruitment and transport of lysosomal hydrolases to the lysosome; therefore, the disruption of this function may lead to lysosomal dysfunction (Gan-Or et al., 2015). RAB7L1 thus functions downstream of GAK in the retromer sorting process (Hoang, 2014). The authors also suggested an epistatic effect, with nonlinear increase in PD susceptibility when both risk alleles are present in the patient's DNA sequence (MacLeod et al., 2013).

Less is known about NUCKS1 and its involvement in PD pathogenesis. NUCKS1 is a nuclear protein containing several consensus phosphorylation sites for casein kinase II and cyclin-dependent kinases of unknown function (Satake et al., 2009). According to some studies, NUCKS1 might also be involved in inflammation and immunity (Dzamko et al., 2015). The recent GWAS meta-analysis has reported that rs823118 was tagged an expression quantitative trait locus and also a methylation quantitative trait locus for both RAB7L1 and NUCKS1 (Wang et al., 2016).

#### LRRK2

*LRRK2* (leucine-rich repeat kinase 2) gene was first described as a risk factor for the familial form of PD, but was later also confirmed as sporadic PD susceptibility gene by GWASs. It encompasses rare highly penetrant variants as well as more common variants with a lower effect (van der Brug et al., 2015). *LRRK2* mutations are the most common gene defect in sporadic PD (Perrett et al., 2015). The first GWAS that confirmed *LRRK2* as a risk factor for sporadic PD was performed by Satake et al. (2009). Later, its association with PD was also confirmed in other GWASs and their meta-analyses (Do et al., 2011; Nalls et al., 2011, 2014; Lill et al., 2012; Vacic et al., 2014). Six different SNPs were found by GWASs as risk factors, but only one (rs1491942) was also replicated in a later study (Lill et al., 2012). *LRRK2* accounts for a large part of risk for sporadic PD in some populations—Ashkenazi Jews, Arabs, East Asians. Coding variability at the *LRRK2* locus explains 10% of PD risk in these populations. In the outbred Europeans, *LRRK2* and *GBA* account for around 8% of risk. Carriers of *LRRK2* mutations usually have Lewy bodies, the minority has also tangles, but some of them have neither (Hardy, 2010).

LRRK2 is a multidomain protein that has a GTPase and kinase function as well as many protein/protein interaction motifs. It is expressed in axons and dendrites in the striatum and the cortex, but expression is low in nigral cell bodies. LRRK2 seems to regulate actin complexes, vesicle trafficking, endosome maturation, cytoskeletal dynamics, and protein translation (Volta et al., 2015). It was detected as a binding partner of the late-endosomal marker Rab7 and also as a binding partner of the lysosomal marker LAMP2A, which generated the idea that it has an important function in the endo-lysosomal pathway. Mutant LRRK2-RAB7 colocalization can lead to a reduced RAB7 function and impaired late endosomal trafficking events. LRRK2 also interacts with RAB7L1 to modify protein sorting. It also binds the PD susceptibility factor GAK in a complex that promotes removal of Golgi derived vesicles by autophagy-dependent mechanisms (Perrett et al., 2015). It is tightly linked to endocytotic and exocytotic processes required for rapid synaptic vesicles and receptor recycling via interaction with clathrin (Volta et al., 2015). It regulates endophilin association to clathrin-coated vesicles through phosphorylation (Drouet and Lesage, 2014). Furthermore, it influences the action of EndoA via phosphorylation, a protein important for endocytic synaptic vesicles recycling (Inoshita and Imai, 2015). Moreover, LRRK2 is involved in mitochondrial membrane maintenance via fusion/fission processes and in lysosomal autophagy and recycling of V-ATPase required for lysosomal acidification (Ryan et al., 2015; Volta et al., 2015). The dysregulation of mitochondrial function can also be caused by the inhibition of the endogenous peroxidase phosphorylation by mutant LRRK2 (Saiki et al., 2012). LRRK2 also has a role in neuroinflammation by increasing the cytokine release from activated primary microglial cells, which results in neurotoxicitiy (Russo et al., 2014; Blesa et al., 2015). Moreover, it interferes with the translation machinery by phosphorylation of several proteins, for example translation repressor protein 4E-BP (Taymans et al., 2015).

Among PD patients having the LRRK2 mutation, the most common one is p.Gly2019Ser (rs34637584). The penetrance is high—50% at the age of 50 and 74% at the age of 79. This mutation is located in the kinase domain resulting in an increase in the activity (Wallings et al., 2015). 1.6% of sporadic PD patients possess this mutation (Satake et al., 2009). Mutated LRRK2 (p.Gly2019Ser) actually binds to outer mitochondrial membrane, which leads to a decrease in mitochondrial membrane potential and to a decrease in the intracellular ATP level. On the contrary, mitochondrial elongation and interconnectivity were elevated (Subramaniam and Chesselet, 2013).

#### INPP5F

Inositol polyphosphate-5-phosphatase F (*INPP5F*), one of the polyphosphoinositide phosphatases, was first described as a PD risk factor in a meta-analysis carried out by Nalls et al. in 2014 (Nalls et al., 2014), which means that only one SNP was found to be associated with PD susceptibility so far. The gene's product is supposed to be involved in the endocytic pathways. It contains a Sac domain, which is involved in the endocytosis of synaptic vesicles (Inoshita and Imai, 2015; Nakatsu et al., 2015). The knowledge about the gene's function is limited and on the basis of only one study pointing out the association, we cannot reach any conclusions as to the main role of the gene in PD pathology and the actual association.

#### BCKDK/STX1B

*BCKDK/STX1B* (branched chain ketoacid dehydrogenase kinase/syntaxin 1B) locus was established as a PD risk factor in GWAS meta-analysis by Nalls et al. Only one SNP (rs14235) is known to be associated with PD, thus replication studies are needed to confirm the actual association (Nalls et al., 2014).

BCKDK is located in the mitochondrial matrix and plays a major role in valine, leucine and isoleucine catabolism. Its function is phosphorylation and thus inactivation of the BCKD. BCKD concentration is the same in all tissues, whereas BCKDK concentration varies. BCKD's level of function is thus regulated by BCKDK (García-Cazorla et al., 2014).

*STX1B* codes for syntaxin 1B, which functions as a synaptic receptor for vesicle transport. It was previously shown to be directly implicated in the process of calcium-dependent synaptic transmission in rat brain, having been suggested to play a role in the excitatory pathway of synaptic transmission (Plagnol et al., 2011). It plays a role in dopamine neurotransmission (Kalia and Lang, 2015).

#### VPS13C

Vacuolar protein sorting 13 homolog C (VPS13C) was shown to be associated with PD as a risk factor only in the meta-analysis by Nalls et al. (2014). First, it was considered only as a risk factor for sporadic PD, but it has recently been discovered that it could also cause the familial autosomal-recessive early-onset form of PD with a very severe course of the disease (Lesage et al., 2016). The gene has two variants, variant 2 being specific to brain tissue (Velayos-Baeza et al., 2004). It functions in the vesicular transport, more specifically endosomal transport pathway (Inoshita and Imai, 2015). Functional studies of the protein are lacking, but a very recent one also suggests a protein quality control function (Yang et al., 2016). More association and functional studies will have to be conducted to be completely confident of the gene's role in PD susceptibility and pathology.

### Lysosomal autophagy

SNPs within loci associated with lysosomal autophagy recognized as PD susceptibility factors by GWASs are listed in Table 4. These genes are playing their part in general lysosomal functions, especially lysosomal autophagy.

**Table 4:** **SNPs within loci associated with lysosomal autophagy recognized as PD susceptibility factors by GWASs**.

**Gene/locus**	**SNP**	**MAF (1000 genomes project)**	**Risk allele: OR**	**Study**
*GBA/SYT11* Ch 1	rs35749011 g.155135036G>A (intergenic variant)	A = 0.0054	A: OR = 1.824	Nalls et al., 2014
	i4000416 g.155205634T>C c.1226A>C p.Asn409Ser (missense variant)	C = 0.0006	C: OR = 4.05	Do et al., 2011
	rs1630500 g.154855055G>A (intergenic variant)	A = 0.0559	OR = 1.75	Vacic et al., 2014
	rs12726330 g.155108167G>A c.-262G>A (splice region variant)	A = 0.0052	OR = 1.71	Pankratz et al., 2012
	rs34372695 g.156030037C>T c.-1155C>T (upstream gene variant)	T = 0.0088	T: OR = 1.47	Nalls et al., 2011
*SCARB2/FAM47E* Ch 4	rs6812193 g.77198986C>T c.871-236C>T (intron variant)	T = 0.3133	C: OR = 1.1	Nalls et al., 2014
			C: OR = 1.19	Do et al., 2011
			C: OR = 1.12	Simon-Sanchez et al., 2009

*Ch, Chromosome. Data compiled from the GWAS catalog database, the dbSNP and the e!Ensembl (Sherry et al., 2001; Welter et al., 2014; Yates et al., 2016)*.

#### GBA/SYT11

GBA (beta acid glucosidase) is associated with PD susceptibility according to several GWASs and their meta-analyses, but the first association was found in 2011 by Do et al. (Do et al., 2011; Pankratz et al., 2012; Nalls et al., 2014; Vacic et al., 2014). On the other hand, *SYT11* (synaptotagmin XI) was confirmed as a PD risk gene by Nalls et al. in 2011 (Nalls et al., 2011, 2014). No SNP has been replicated in a later study.

GBA is located within the inner lysosomal membrane, where it cleaves membrane glucocerebrosides into ceramide and glucose. Functional studies have shown that any kind of reduction in GBA enzymatic activity leads to the accumulation of SNCA which, in turn, inhibits the function of normal GBA, causing the additional aggregation of SNCA. The authors hypothesize that the reduction of GBA function, either caused by *GBA* mutations or impaired GBA trafficking from ER and Golgi to lysosomes, increases the glucocerebrosides concentration in lipid rafts. This change in membrane composition may lead to the reduced formation of LAMP2A (chaperone-mediated autophagy receptor) protein-complexes, which could result in diminished autophagy of SNCA and its accumulation. GBA dysfunction may also lead to general disruption in lysosomal function and autophagy. Furthermore, it can also be the reason for defective mitophagy and mitochondrial damage. It was also shown that PD patients with no mutation in the *GBA* may have a lower enzyme activity. This implies that other factors may also affect the GBA function, for example environmental factors (Gan-Or et al., 2015).

*SYT11* encodes a calcium-sensing protein involved in membrane trafficking in synaptic transmision and is also a substrate of Parkin, which is a risk factor for the familial form of PD. Due to its ability of calcium-dependent phospholipid binding, it is important in the regulation of vesicle fusion and endocytosis at synaptic terminals (Ran and Belin, 2014). More studies will have to be performed in order to properly determine the function of the gene.

#### SCARB2/FAM47E

*SCARB2/FAM47E* (scavenger receptor class B member 2) locus was first found to be associated with PD in 2009 by Simon-Sanchez et al.; this was later confirmed by two other GWASs—Do et al. and Nalls et al. The same SNP (rs6812193) was pointed out in all three studies (Simon-Sanchez et al., 2009; Do et al., 2011; Nalls et al., 2014).

*SCARB2* encodes the lysosomal membrane protein type 2 (LIMP-2), which is a GCase receptor. It directs GBA to lysosomes. The reduced function of SCARB2 can lead to a reduced GBA activity and decreased SNCA degradation (Gan-Or et al., 2015). The receptor, being an enterovirus 71 receptor, also plays a role in the neuroinflammation process (Dzamko et al., 2015). *FAM47E* lies in close proximity to *SCARB2* and the most frequently associated SNP (rs6812193) is situated between the two genes. However, among the two, SCARB2 seems to be a more promising candidate for a PD risk factor (Do et al., 2011; Ran and Belin, 2014).

### Immune system

SNPs within loci associated with the immune system recognized as PD susceptibility factors by GWASs are listed in Table 5. The genes' function in PD susceptibility and pathology should be tested in a more targeted way with the help of cell and animal models. There are some indications that genes mentioned below play a role in the immune system, but we do not know to what extent and if this is their main purpose, except for *HLA-DQB1* whose function is quite well determined. There is also a need for more association studies especially for *STK39, HLA-DQB1* and *DDRGK1*.

**Table 5:** **SNPs within loci associated with immune system recognized as PD susceptibility factors by GWASs**.

**Gene/locus**	**SNP**	**MAF (1000 genomes project)**	**Risk allele: OR**	**Study**
*BST1* Ch 4	rs11724635 g.15737101C>A (intron variant)	A = 0.4079	A: OR = 1.126	Nalls et al., 2014
			A: OR = 1.15	Nalls et al., 2011
	rs4538475 g.15737937A>G c.^*^195A>G (intron variant)	G = 0.3089	OR = 1.24	Satake et al., 2009
	rs4698412 g.15737348G>A c.852-328G>A (intron variant)	A = 0.4077	OR = 1.14	Pankratz et al., 2012
			A: OR = 1.14	Saad et al., 2011
*STK39* Ch 2	rs1474055 g.169110394C>T (intergenic variant)	T = 0.2007	T: OR = 1.214	Nalls et al., 2014
	rs2102808 g.169117025G>T (intergenic variant)	T = 0.2083	C: OR = 1.18	Nalls et al., 2011
*HLA-DQB1* Ch 6	rs9275326 g.32666660C>T (intergenic variant)	T = 0.0843	C: OR = 1.21	Nalls et al., 2014
*DDRGK1* Ch 20	rs8118008 g.3168166A>G (downstream gene variant)	G = 0.4597	A: OR = 1.111	Nalls et al., 2014

*Ch, Chromosome. Data compiled from the GWAS catalog database, the dbSNP and the e!Ensembl (Sherry et al., 2001; Welter et al., 2014; Yates et al., 2016)*.

* *Denotes SNPs in 3'-UTR*.

#### BST1

*BST1* (bone marrow stromal cell antigen-1) was recognized as a PD risk factor in the Satake et al. GWAS in 2009 and after that in several other GWASs. Three intron variants were found to be associated with PD risk. Two of them (rs11724635 and rs4698412) were replicated in a later study (Satake et al., 2009; Nalls et al., 2011, 2014; Saad et al., 2011; Pankratz et al., 2012). BST1, a member of the CD38 gene family, is a cell surface protein bound to the membrane by glycosylphosphatidylinositol linkage; it possesses both ADP ribosyl cyclase and cyclic ADP ribose hydrolase enzymatic activities. It produces cyclic ADP-ribose, which as a second messenger releases calcium from intracellular ER stores, leading to calcium influx. Calcium is a great regulator of neurotransmitter release from presynaptic terminals; therefore, a disruption in neuronal calcium homeostasis could lead to selective death of dopaminergic neurons. Interestingly, BST1 seems to be an important factor in the immune system, since it is highly expressed in bone marrow cells in patients with rheumatoid arthritis and facilitates immature B-cell proliferation and growth (Chen et al., 2014; Ran and Belin, 2014).

#### STK39

*STK39* (serine threonine kinase 39) is highly expressed in the brain and pancreas (Wang et al., 2014). It codes for a SPAK protein (Ste20-related proline/alanine rich-kinase) (Kumaran and Cookson, 2015). As a PD risk factor, it was first described by Nalls et al. in 2011 and then again confirmed three years later by the same group. Two different intergenic variants were pointed out in these studies (Nalls et al., 2011, 2014). It is a serine/threonine kinase, which is activated under cellular stress and plays a role in stress signals, ion homeostasis and inflammation (Li et al., 2013; Ran and Belin, 2014). It has been shown that its overexpression alters intestinal inflammatory levels in mouse models of colitis, whereas its knockout attenuates intestinal inflammation (Dzamko et al., 2015; Kumaran and Cookson, 2015). Apart from PD, it has been associated with hypertension, autism and early-stage non-small-cell lung cancer (Li et al., 2013).

#### HLA-DQB1

*HLA-DQB1* (major histocompatibility complex, class II, DQ beta 1) was shown to be associated with the risk for PD in the Nalls et al. study in 2014 (Nalls et al., 2014). Only one intergenic variant was shown to be associated with a PD risk, which means that more association studies confirming the association need to be conducted. It plays a key role in the immune system by presenting peptides to the antigen presenting cells. The HLA region is one of the most complex regions in the genome. It encompasses a high number of closely packed genes, with numerous polymorphisms and complicated patterns of linkage disequilibrium. As part of immune response HLA genes, are involved in many pathologies, such as autoimmune diseases, infections and malignant and neurological disorders. The HLA region is divided into classes, I and II, but they both code for proteins that present antigens to T cell receptors as part of the adaptive immune system. HLA class II molecules are expressed in antigen presenting cells—macrophages, B lymphocytes and dendritic cells. They consist of two chains, alpha (DQA) and beta (DQB) (Lampe et al., 2003; Wissemann et al., 2013; Chang et al., 2015). Several *HLA* genes (Hill-Burns et al., 2011) are thought to be associated with PD, besides *HLA-DQB1* also *HLA-DRA* (Hamza et al., 2010; Pankratz et al., 2012; Hill-Burns et al., 2014) and *HLA-DRB* (Ahmed et al., 2012).

#### DDRGK1

*DDRGK1* (DDRGK domain containing 1) gene was found to be associated with PD in the meta-analysis of GWASs performed by Nalls et al. (2014), but the association has not been confirmed yet. DDRGK1 regulates the nuclear factor-κB (NF-κB) activity (Dzamko et al., 2015). NF-κB is a group of inducible nuclear transcription factors; it is largely expressed in a wide variety of cells and regulates the expression of a lot of genes in response to different stimuli. It plays an important role in inflammation, immune system as well as in tumorigenesis (Mitchell et al., 2016). DDRGK1 is localized in the endoplasmic reticulum (ER) and its expression is induced by ER stress (Xi et al., 2013).

### Neurodevelopment, neuron cell differentiation and survival

SNPs within loci associated with neurodevelopment, neuron cell differentiation and survival recognized as PD susceptibility factors by GWASs are listed in Table 6.

**Table 6:** **SNPs within loci associated with neurodevelopment, neuron cell differentiation and survival recognized as PD susceptibility factors by GWASs**.

**Gene/locus**	**SNP**	**MAF (1000 genomes project)**	**Risk allele: OR**	**Study**
*CCDC62* Ch 12	rs11060180 g.123303586A>G c.2002-4327A>G (intron variant)	G = 0.2516	A: OR = 1.105	Nalls et al., 2014
	rs12817488 g.123296294G>A c.1852-1523G>A (intron variant)	A = 0.4159	T: OR = 1.14	Nalls et al., 2011
*RIT2* Ch 18	rs12456492 g.43093415A>G c.103+22002T>C (intron variant)	G = 0.3297	G: OR = 1.11	Nalls et al., 2014
			OR = 1.19	Pankratz et al., 2012
	rs4130047 g.43098270T>C c.103+17147A>G (intron variant)	C = 0.3237	C: OR = 1.16	Do et al., 2011
*FGF20* Ch 8	rs591323 g.16697091G>A (intron variant)	A = 0.3652	G: OR = 1.09	Nalls et al., 2014
*GCH1* Ch 14	rs11158026 g.55348869C>T c.344-16715G>A (intron variant)	C = 0.4898	C: OR = 1.11	Nalls et al., 2014
*GPNMB* Ch 2	rs199347 g.23293746A>G c.224-42A>G (intron variant)	A = 0.4832	A: OR = 1.11	Nalls et al., 2014

*Ch, Chromosome. Data compiled from the GWAS catalog database, the dbSNP and the e!Ensembl (Sherry et al., 2001; Welter et al., 2014; Yates et al., 2016)*.

#### CCDC62

*CCDC62* (coiled-coil domain containing 62) was found to be related to PD susceptibility in the Nalls et al. studies conducted in 2011 and 2014 (Nalls et al., 2011, 2014) pointing out two different intron variants. The gene has a role in cell growth, estrogen receptor transactivation, cyclin D1 expression in prostate cancer cells as well as in other varieties of cancer, since its antibodies are often produced and thus detected (Li et al., 2013; Lu et al., 2016). Until recently, it was mainly reported as related to cancer (Yu et al., 2015). In order to define the gene's function more precisely, more functional studies are required.

#### RIT2

*RIT2* (ras-like without CAAX 2) was first described as a PD susceptibility factor in the GWAS performed by Do et al. in 2011 and after that confirmed in two meta-analyses (Pankratz et al., 2012; Nalls et al., 2014). Two variants within the gene were found to be associated with the PD risk. One of them (rs12456492) was replicated, while the other (rs4130047) was significant in only one study (Do et al., 2011; Pankratz et al., 2012; Nalls et al., 2014). RIT2 is a small GTPase of the Ras family. It is neuron-specific and preferentially expressed in dopaminergic neurons in substantia nigra (Ran and Belin, 2014). Accumulating reports suggest its important role in neuronal differentiation and function. It promotes neurite outgrowth through Rac/Cdc42 activation and calmodulin association (Zhang et al., 2013). It binds calmodulin 1, a phosphorylase kinase, in a calcium-dependent manner and regulates certain signaling pathways and cellular processes (Do et al., 2011). It interacts with SNCA and tau via calmodulin (Lu et al., 2015). RIT2 is also a specific interacting partner of the dopamine transporter (DAT). DAT is a transmembrane protein which can be internalized by protein kinase C-mediated endocytosis and thus downregulated. It was proposed that extracellular dopamine concentrations and half-life are regulated in this manner. This process presumably depends on RIT2 GTPase activity (Zhang et al., 2013; Ran and Belin, 2014). A reduced production of RIT2 was detected in postmortem samples of PD patients when compared to controls (Bossers et al., 2009). In another postmortem study, a possible regulation of INF-γ signaling by RIT2 was discovered (Liscovitch and French, 2014).

#### FGF20

*FGF20* (fibroblast growth factor 20) was proved to be associated with PD in the Nalls et al. meta-analysis (Nalls et al., 2014), in which one intron variant within the gene with a positive association was found. More studies will have to be done to confirm this association. FGF20 is a neurotrophic factor preferentially expressed in substantia nigra pars compacta. It acts in an autocrine/paracrine manner. FGF20 regulates central nervous development and function (Plagnol et al., 2011). It plays a major role in dopaminergic neurons differentiation and survival (Itoh and Ohta, 2013). According to some studies, FGF20 also increases SNCA levels in dopaminergic neurons, but there is a huge discrepancy among studies (Wang et al., 2008; Wider et al., 2009; Sekiyama et al., 2014; Tarazi et al., 2014).

#### GCH1

*GCH1* (GTP cyclohydrolase 1) was found to be associated with PD pathology only in the Nalls et al. meta-analysis of GWASs (Nalls et al., 2014), which means that more studies whose results will show a positive association are required to claim that the association is in fact true. GCH1 is an essential enzyme in dopamine synthesis in the nigrostriatal nervous cells. Mutations in this gene can result in the degeneration of nigral neurons. Loss of function leads to severe depletion of dopamine levels and is the most frequent cause of DOPA-responsive dystonia (DRD), a rare disease that classically occurs during the childhood and is manifested as generalized dystonia and an excellent sustained response to low doses of levodopa, usually without motor fluctuations. DRD is often associated with PD. Mutations in the gene might cause striatal cell death and thus evolve into PD. The enzyme controls the first and rate-limiting step in the biosynthesis of tetrahydrobiopterin (BH_4_), which is a cofactor of tyrosine hydroxylase, which converts tyrosine to levodopa (Mencacci et al., 2014; Rengmark et al., 2016). It may also have some role in inflammation (Dzamko et al., 2015). The debate on whether this gene is related to the familial or sporadic form is still ongoing. Some say that PD-like symptoms in adulthood could also be a different phenotype of DRD.

#### GPNMB

*GPNMB* (glycoprotein nonmetastatic melanoma protein B) was recognized as associated with a PD risk in the Nalls et al. meta-analysis in 2014. As the association was found in one study only, we cannot conclude that the association is definite (Nalls et al., 2014). It presumably plays an important role in neuronal survival and neuroprotection (Xu et al., 2016). It is also known as osteoactivin, dendritic cell-heparin integrin ligand or haematopoietic growth factor inducible neurokinin-1 type. It is important for the differentiation and functioning of osteoclasts and osteoblasts, the impairment T-cell activation, the invasion, and metastasis of many cancers and the regulation of degeneration/regeneration of extracellular matrix in skeletal muscles. The gene was also associated with amyotrophic lateral sclerosis as another type of a neurodegenerative disease (Tanaka et al., 2012). The protein mostly localizes in lysosomes. It is also involved in phagocytosis and helps to recruit an autophagy protein LC3-II to the phagosome. It is essential for the fusion of phagosome and lysosome to degrade the phagosome content (Gan-Or et al., 2015). GPNMB is somehow involved in innate and adaptive immunity along with many PD susceptibility factors. It may play a role in regulating microglial inflammation downstream of LPS activation (Dzamko et al., 2015; Herrero et al., 2015).

### Mitochondrial homeostasis

SNPs within loci associated with mitochondrial homeostasis recognized as PD susceptibility factors by GWASs are listed in Table 7.

**Table 7:** **SNPs within loci associated with mitochondrial homeostasis recognized as PD susceptibility factors by GWASs**.

**Gene/locus**	**SNP**	**MAF (1000 genomes project)**	**Risk allele: OR**	**Study**
*SREBF1/RAI1* Ch 17	rs11868035 g.17715101G>A c.^*^835C>T (splice region variant)	G = 0.4876	G: OR = 1.18	Do et al., 2011
*MCCC1* Ch 3	rs12637471 g.182762437G>A c.732+764C>T (intron variant)	A = 0.3375	G: OR = 1.1876	Nalls et al., 2014
	rs11711441 g.182821275G>A (intron variant)	A = 0.1406	G: OR = 1.19	Nalls et al., 2011
	rs10513789 g.182760073T>G c.733-535A>C (intron variant)	G = 0.3115	T: OR = 1.25	Do et al., 2011

*Ch, Chromosome. Data compiled from the GWAS catalog database, the dbSNP and the e!Ensembl (Sherry et al., 2001; Welter et al., 2014; Yates et al., 2016)*.

* *Denotes SNPs in 3'-UTR*.

#### SREBF1/RAI1

*SREBF1/RAI1* (sterol regulatory element binding transcription factor 1/retinoic acid induced 1) locus was associated with PD susceptibility for the first time by Do et al. GWAS (Do et al., 2011). In 2014, it was again confirmed by the meta-analysis of GWASs (Nalls et al., 2014). The same SNP (rs11868035) was pointed out in both studies.

*SREBF1* encodes SREBP-1 (sterol regulatory element-binding protein 1), a transcriptional activator (Do et al., 2011). The connection of mitophagy pathway and familial PD has been firmly established via two genes—*PINK1* and *Parkin*, but also GWASs and functional studies found the association between this pathway and sporadic PD through *SREBF1*. It is involved in the mitophagy as well as in the regulation of lysosomal lipid accumulation. Mitochondria and lipid metabolism have complementary functions via Krebs cycle. Knockdown of SREBF1 blocks the translocation of Parkin into the mitochondria and consequently decreases mitophagy (Gan-Or et al., 2015). This process can be restored by the addition of exogenous lipids, both fatty acids and cholesterol. Downregulation of *SREBF1* may also decrease mitophagy by blocking the stabilization of PINK1 on the outer mitochondrial membrane of injured mitochondria, which can also be repaired by additional exogenous lipids (Ivatt and Whitworth, 2014). It regulates cholesterol synthesis and its cellular uptake from plasma LDL. A reduced expression of SREBF1 also downregulates the *NPC1* gene, which may lead to the accumulation of cholesterol within late endosomes and lysosomes (Gan-Or et al., 2015). Autophagic turnover of mitochondria needs to be balanced with its biogenesis, which heavily relies upon SREBP pathway for membrane synthesis (Ivatt et al., 2014). Some studies also suggest that SREBP-1 is an important mediator of NMDA-induced excitotoxicity (Do et al., 2011). It may also be involved in the innate immune response via lipid metabolism (Jeon and Osborne, 2012).

RAI1 is a part of transcription regulation mechanism, beacuse it has the ability to remodel chromatin and interacts with the basic transcriptional machinery (Do et al., 2011).

#### MCCC1

*MCCC1* [methylcrotonyl-CoA carboxylase 1 (alpha)] was shown to be associated with sporadic PD in 2011 with two GWASs and after that again confirmed with the meta-analysis. In all three studies, a different intron variant was implicated as a risk factor (Do et al., 2011; Nalls et al., 2011, 2014). Its product, a biotin-requiring enzyme, functions within the mitochondria as a part of leucine catabolism pathway (Wang et al., 2014). It is currently not known if this enzyme has a role in the mitophagy or mitochondrial quality control. Researchers are also debating on whether this gene or rather *LAMP3* located on the same locus is involved in PD pathogenesis (Gan-Or et al., 2015).

### Other processes

SNPs within loci associated with other processes recognized as PD susceptibility factors by GWASs are listed in Table 8. More association and functional studies will have to be performed to discover a clear connection between a PD risk and genes mentioned below.

**Table 8:** **SNPs within loci associated with other processes recognized as PD susceptibility factors by GWASs**.

**Gene/locus**	**SNP**	**MAF (1000 genomes project)**	**Risk allele: OR**	**Study**
*ACMSD/TMEM163* Ch 2	rs6430538 g.135539967C>T (intron variant)	C = 0.1891	C: OR = 1.1429	Nalls et al., 2014
			OR = 1.15	Pankratz et al., 2012
	rs6710823 g.135592381G>A (intron variant)	NA	G: OR = 1.1	Nalls et al., 2011
*MIR4697* Ch 11	rs329648 g.133895472T>C (downstream gene variant)	T = 0.4645	T: OR = 1.105	Nalls et al., 2014
*SIPA1L2* Ch 1	rs10797576 g.232664611C>T (intron variant)	T = 0.1274	T: OR = 1.131	Nalls et al., 2014

*NA, data not available; Ch, Chromosome. Data compiled from the GWAS catalog database, the dbSNP and the e!Ensembl (Sherry et al., 2001; Welter et al., 2014; Yates et al., 2016)*.

#### ACMSD/TMEM163

*ACMSD/TMEM 163* (aminocarboxymuconate semialdehyde decarboxylase-transmembrane protein 163) was shown to be associated with PD by Nalls et al. studies as well as by Pankratz et al. meta-analysis of GWASs. Two variants were found in these three studies, which means that one of them (rs6430538) was also replicated (Nalls et al., 2011, 2014; Pankratz et al., 2012). This gene is expressed at very low but significant levels in the brain and encodes a cytosolic enzyme (Martí-Massó et al., 2013). ACMSD is involved in the picolinic and quinolinic acid homeostasis and is thus a possible therapeutic target for many central nervous system disorders (Plagnol et al., 2011). ACMSD is also categorized as a detoxifying enzyme because of its ability to prevent the accumulation of neurotoxic metabolite quinolinate, which is one of the tryptophan catabolites (Ran and Belin, 2014). It also participates in the kynurenine pathway of tryptophan catabolism. Mutations in the gene presumably result in a decreased enzymatic activity, which means that the conversion of tryptophan to picolinic acid is blocked. Consequently, quinolinic acid levels in the brain are elevated. Quinolinat is also an intermediate of the de novo synthesis of NAD from tryptophan (Martí-Massó et al., 2013).

#### MIR4697

*MIR4697* is a non-protein coding sequence that was shown to be associated with PD in the Nalls et al. meta-analysis in 2014 (Nalls et al., 2014) in which one SNP with a positive association was found. The function of the microRNA in general is not known, much less its function in PD pathology (Chen et al., 2016).

#### SIPA1L2

*SIPA1L2* (signal-induced proliferation-associated 1 like 2) is a gene related to PD pathogenesis according to the Nalls et al. study conducted in 2014, but the function of the gene has to be determined yet. Only one SNP has been pointed out as a risk factor so far (Nalls et al., 2014).

## Applicability of the knowledge about genetic susceptibility factors for PD

The knowledge about the genetic factors of PD risk or pathogenesis related to a specific pathway could be applied in the clinical setting to support early diagnosis and to predict disease prognosis as suggested by the study of Nalls et al. (2015). Furthermore, the information on susceptibility genes could be easily integrated in composite diagnostic and/or prognostic algorithms that at present include clinical characteristics and imaging data (Siderowf et al., 2012; Gaenslen et al., 2014; Noyce et al., 2014). These genetic factors could also help to stratify the patients on the basis of underlying molecular defect into respective groups that would benefit from targeting a specific pathway with novel or existing treatment approaches, which would enable personalized treatment planning. The latter also indicates an opportunity to identify novel potential targets for innovative treatment approaches and supportive measures.

First, described genetic factors could support early diagnosis. This is an important goal for clinicians dealing with PD, because it is important to start treatment early in the disease course (Olanow and Schapira, 2013). It would be ideal to identify patients in the prodromal phase of the disease (Berg et al., 2015). By the time motor symptoms occur, more than half of dopaminergic neurons are already lost. The time of the prediagnostic phase, which can last for up to 20 years, thus presents the window of opportunity for searching for and for the application of neuroprotective treatment (Salat et al., 2016). For the purpose of early diagnosis, a panel of genes should be tested for different mutations and polymorphisms to check the cumulative effect of the genetic defects as a single mutation or polymorphism has a very low contribution to disease risk. The combined assessment of early non-motor symptoms with a genetic predisposition could be an ideal method to identify the patients in the earliest phases of the disease. Only genetic testing for diagnosis of sporadic PD or for the prediction of developing sporadic PD is not specific and sensitive enough at the moment. There is no known genetic factor or combination of genetic factors that could conclusively predict the development of sporadic PD. Therefore, genetic testing would probably be more suitable for high risk families with positive PD history or for people with manifestations of early non-motor symptoms and not for general population. Furthermore, a cumulative score and not individual genetic factors should be considered, jointly with clinical and imaging data. Nalls et al. developed an algorithm for early diagnosis of PD on the basis of clinical and genetic classification. Their model included several clinical factors, such as age, gender, olfactory function and family history of PD. They combined clinical factors with a genetic risk score, that accounted for 28 genetic variants identified and replicated in the most recent large-scale meta-analysis of PD GWASs data plus two other rare risk variants, a *GBA* (rs76763715) and a *LRRK2* (rs34637584) mutation. The model correctly distinguished between patients with PD and controls with an area under curve of 0.923 with both high sensitivity (0.834) and specificity (0.903). They also validated the model on five other cohorts and AUC was never lower than 0.894 (Nalls et al., 2015). There are a few prospective longitudinal studies running (PARS, TREND, PREDICT-PD), which are looking for biomarkers to identify PD and predict the way of disease progression before a diagnosis based on motor symptoms can be made, and assessing combinations of risk factors and early features of PD. Among these studies, PARS included a cohort of elderly patients with olfactory dysfunction, TREND included people older than 50 years with one of prodromal symptoms of PD (olfactory dysfunction, REM sleep behavior disorder or depression), while PREDICT-PD included general population from 60 to 80 years of age irrespective of PD prodromal symptoms (Salat et al., 2016).

Furthermore, the impact of genetic susceptibility factors on the disease prognosis could be evaluated. It would be interesting to explore the association between the course of the disease and the genetic defect in genes involved in certain pathways. For example, *GBA* mutations or *MAPT* H1 allele status might be independent risk factors for cognitive impairment in PD patients and the knowledge of these allele statuses in patients could have an impact on the treatment plan (Clarimón and Kulisevsky, 2013).

Moreover, genetic susceptibility factors segregating in different pathways could help to stratify PD patients into distinct groups according to the differences in the main molecular cause of the disease. So far, the stratification of patients has only been made based on different phenotypes (van Rooden et al., 2010, 2011; Fereshtehnejad et al., 2015), but our main idea is to group them on the basis of the main molecular cause of the disease rather than on the basis of different ways of manifestation. The stratification should be based on cumulative effects of genetic susceptibility factors within a particular pathway as well as across pathways, so different combinations of genetic defects should be tested to find a way to stratify the PD patients. This approach was already addressed in a review written by Schapira et al. (Schapira, 2013), where the problem of aetiologically heterogeneous cohorts of PD patients evaluated in different studies was exposed. The latter makes it difficult to seek for different genetic biomarkers as there may be different ones in distinct groups. The stratification of PD patients based on underlying genetic defects could also be beneficial in a clinical setting as patients with different genetic defects may need different treatment plans. This kind of personalized treatment could become the treatment of choice in PD in the future, as we could stratify patients into groups according to their compromised pathway and treat them according to their underlying pathogenic processes. The treatment could be adjusted for each group to obtain the best possible response.

Last but not least, the disease genes or the products of the genes involved in the aethiopathogenesis could serve as novel targets for neuroprotective or disease-modifying drugs to delay or prevent disease progression. However, the problem of a very modest impact of the above-mentioned mutations and polymorphisms on the PD pathology occurs. If the impact of a single mutation is very low, there is probably no point in developing a new active pharmaceutical agent specific for that target, because the effect on the disease course would probably not be satisfactory. A more feasible way of incorporating means of personalized medicine and patient startification into PD management is to target a corrupted pathway as a whole instead of focusing only on the compromised gene and its product. The goal would be to strengthen or improve the function of a compromised pathway, such as protein aggregation; protein and membrane trafficking; lysosomal autophagy; immune system; neurodevelopment, neuron cell differentiation and survival; mitochondrial homeostasis; and genes involved in other processes. Although this is a less specific approach, it focuses on the putative main cause of the disease occurrence in a particular patient. In the light of this, there is a possibility of the repurposing of drugs already used for other indications, for example ursodeoxycholic acid, nicotine, caffeine, isradipine, exenatide, statins (Salat et al., 2016). In order to establish such a pathway-oriented approach, clinical trials must focus on PD patients with the same underlying molecular defect. As long as heterogenous cohorts are evaluated in these studies, no such treatment will be available (Korczyn and Hassin-Baer, 2015).

## Future perspectives

With the emergence of GWASs, researchers believed that the discovery of the exact genetic background of PD is at their fingertips, but more questions arose afterwards. GWASs are a type of studies with no hypothesis at the time of their performance, so the results must be critically evaluated and supported by functional studies of genes recognized to be involved in the pathogenesis of a certain disease. Apart from functional studies of not yet known genes' functions, animal disease models should also be investigated to check and validate the results of GWASs. We also have to be aware of the fact, that GWAS usually detect common SNPs that most probably have a modest impact only. Rare but highly penetrant SNPs are often overlooked. Hence, in terms of susceptibility prediction or early diagnosis, physicians should check several genetic biomarkers (SNPs) and their cumulative impact on the prognosis along with clinical and imaging data. Additionally, SNPs associated with a certain disease are often not located within the disease gene, but are rather in linkage disequlibrium with it.

The main goal of searching for genetic biomarkers of PD susceptibility is early diagnosis or more optimistically, a chance to slow down or even prevent the development of the disease. Furthermore, with better characterization of risk genes' functions, we could stratify the PD patients into groups according to the main route of pathogenesis. Prospective monitoring is needed to compare the symptomatology as well as the rate of progression between the groups in order to get a chance of tailoring the therapy to each group. To take this idea to the next level, one could also find new drug targets based on the susceptibility genes or their products if the impact of the susceptibility gene is high enough. A personalized approach would allow to treat each group of patients with the most effective and safest drug and adopt optimal supportive measures, e.g., antiinflammatory therapy for patients with an increased inflammatory response.

## Conclusions

In conclusion, the newest GWASs have identified top hit susceptibility genes with emerging information on their physiological functions and involvement in PD pathology. These susceptibility genes belong to specific pathways that are already known to be compromised in PD and could thus serve as a genetic tool for the stratification of patients. In our opinion, this would allow the treatment of patients according to the underlying cause of their clinical signs to choose the most beneficial treatment with minimal side effects. This knowledge gives the opportunity to personalize the treatment of PD patients, but more studies need to be carried out on cell models, animal models and patients before new knowledge can be translated into the everyday clinical practice of PD treatment.

## Author contributions

All authors have made a substantial intellectual contribution to this work and approved its final version for submission. VD and SR formed the review focus. SR conducted the literature review and summarized and wrote the first draft of the manuscript under the supervision of VD. VD and MT evaluated the manuscript and contributed to the final version.

## Funding

This work was partially funded by the Horizon 2020 Artemida 664536 Grant and the Slovenian Research Agency (ARRS) Grant P1-0170.

### Conflict of interest statement

The authors declare that the research was conducted in the absence of any commercial or financial relationships that could be construed as a potential conflict of interest.
